# The association between mid‐life personality traits and late‐life dementia risk in the Wisconsin Longitudinal Study

**DOI:** 10.1002/alz.092932

**Published:** 2025-01-09

**Authors:** Brittani J. Strait, Ralph Trane, Kate Lange, Sanjay Asthana, Victoria J. Williams

**Affiliations:** ^1^ University of Wisconsin‐Madison, Madison, WI USA

## Abstract

**Background:**

Personality traits, namely higher neuroticism and lower conscientiousness, are predictive of dementia, although the complex relationship has yet to be fully explained. With >60 years of prospectively collected data, the Wisconsin Longitudinal Study (WLS) provides a unique opportunity to thoroughly evaluate this association in a population‐based randomly selected cohort. Using a gold‐standard diagnostic approach to characterize dementia prevalence, we leverage life course WLS data to examine the association between mid‐life personality traits and late‐life dementia risk.

**Method:**

Big Five personality traits (neuroticism, conscientiousness, openness, extraversion, and agreeableness) were prospectively measured in 1993‐94 at mid‐life (ages 54‐55). Cognitive status was determined in late‐life starting in 2020 (age 81) using a two‐phased approach. Participants were first screened via the Telephone Interview for Cognitive Status (TICS‐m), with those scoring below cutoff (≤28) selected for additional comprehensive medical and cognitive assessment reviewed by a multi‐disciplinary clinician consensus panel to determine dementia diagnosis. To evaluate the association between midlife personality traits and late‐life all‐cause dementia risk, we performed a binary logistic regression controlling for age, sex, ApolipoproteinE‐4 (ApoE‐4) status, adolescent IQ, education, comprehensive smoking history, and lifetime burden of systemic health factors (hypertension, diabetes, stroke, heart disease).

**Result:**

3765 WLS participants were included in analyses. The overall logistic regression model was statistically significant (Chi^2^(14) = 207.52, p<0.001), explaining 12.3% of the variance in dementia outcome. Each unit increase in mid‐life conscientiousness corresponded to a 4.5% decrease in the odds of late‐life dementia (OR = 0.955 [95% CI: 0.926‐0.985], p = 0.003), while the remaining four personality factors were not predictive of dementia risk.

**Conclusion:**

Using a longitudinal population‐based cohort, we find that lower mid‐life conscientiousness increases risk for dementia in late‐life, and conversely, higher mid‐life conscientiousness is protective for late‐life dementia. Although prior research suggests higher neuroticism is also associated with dementia outcomes, our data did not support this finding. Future work should explore potential pathways and mechanisms by which personality traits affect cognition and neurodegenerative disease risk, including the possible mediating role of personality‐associated health behaviors.

